# Exploring a pilot alcohol and other drug (AOD) nurse practitioner mentoring program: Empirical research mixed methods: A pilot nurse practitioner mentoring program

**DOI:** 10.1002/nop2.2250

**Published:** 2024-08-09

**Authors:** Adam Searby, Dianna Burr, Colleen Blums, Jason Harrison, Darren Smyth

**Affiliations:** ^1^ Monash University School of Nursing and Midwifery Clayton Victoria Australia; ^2^ Drug and Alcohol Nurses of Australasia (DANA) Brisbane Queensland Australia; ^3^ Central Queensland Mental Health Alcohol and Other Drug Service Rockhampton Queensland Australia; ^4^ Queensland Injectors Health Network (QuIHN) Gold Coast Queensland Australia

**Keywords:** clinical mentoring, continuing professional development, mentoring, nurse practitioners, nurses

## Abstract

**Aim:**

To explore the effectiveness and acceptability of a pilot mentoring program for alcohol and other drug (AOD) nurse practitioners (also known globally as addiction nurse practitioners).

**Design:**

Mixed method evaluation.

**Methods:**

Two‐phase evaluation comprising survey (demographics, pre‐ and post‐program perceived competency and confidence) with 15 participants completing the pre survey and 10 participants completing the post survey, and qualitative interviews after the program with 10 participants.

**Results:**

The quantitative results indicate statistically significant increases in some domains of perceived competence and confidence in treatment. Qualitative findings indicate that participants valued peer support and mentoring from experienced nurse practitioners. Where formal residency or internship programs for nurse practitioners do not exist, informal mentoring programs may address issues inherent in nurse practitioner transition that may impact retention. We recommend further exploration of mentoring programs with larger sample sizes to determine if self‐reported clinical improvements are noted.

**Implications for Profession and/or Patient Care:**

Nurse practitioners are a vital part of the healthcare system; their advanced skills and knowledge place them in an ideal position to address prescriber shortages and access to care for populations underserved by healthcare. However, literature indicates that they are often underutilised, and transition to autonomous practice remains a challenge. Our exploration of a pilot mentoring program for nurse practitioners shows that their knowledge and perceived skills are high, yet peer assistance is valued in transitioning from advanced practice registered nurse to autonomous nurse practitioner. We recommend further trialling and evaluation of nurse practitioner mentoring programs to both increase supply of nurse practitioners and provide greater access to quality healthcare for underserved populations.

**Impact:**

**What problem did the study address?**

The ability of nurse practitioners to offer advanced practice interventions such as diagnosis and medication management potentially provides a solution to healthcare resource shortages. However, current literature indicates that advanced nurses transitioning to nurse practitioner roles suffer transition shock, leading to burnout and poor retention.

**What were the main findings?**

Although this pilot mentoring program shows significant improvements in survey responses on confidence and capability, qualitative data shows that neophyte nurse practitioners value peer support and mentoring from more experienced practitioners. Participants described maintaining ongoing connections with both mentors and fellow mentees, which aided transition to the role of autonomous nurse practitioner.

**Where and on whom will the research have an impact?**

The results of this pilot mentoring program indicate that there is benefit to these programs for neophyte nurse practitioners in many specialties. This paper indicates that mentoring programs for nurse practitioners may provide a community of practice and may have a positive impact on transition shock.

**Reporting Method:**

Good Reporting of a Mixed Methods Study (GRAMMS) checklist.

**Patient or Public Contribution:**

No patient or public contribution.

## 1 INTRODUCTION

In Australia, the role of nurse practitioner is in its relative infancy, emerging in 2000 after legislation allowing nurse practitioners to practice under the federally regulated healthcare legislation system was introduced in 1998 (MacLellan et al., 2017). Globally, advanced nursing roles and the role of nurse practitioner have existed for longer, particularly in North America where these roles were piloted in the 1970s (Sheer & Wong, 2008). Regardless of the country where nurse practitioners are situated, the literature indicates that the role is often underutilised (Currie, 2021; Harvey, 2011; Wu & Darracq, 2020), despite evidence that the nurse practitioner model can achieve clinical outcomes comparable to other healthcare professionals, often at a comparable or lower cost, with greater patient engagement in care (Htay & Whitehead, 2021; Laurant et al., 2018).

Since their introduction in Australia, mental health nurse practitioners have previously been defined as a priority focus for state governments (Fisher, 2005), however the potential for nurse practitioners in alcohol and other drug (AOD) treatment services is only starting to be realised. In these settings, AOD nurse practitioners address prescriber shortages inherent in the Australian system, provide holistic care for mental and physical ill health and an ability to address psychosocial issues occurring co‐morbidly with AOD use (Jones et al., 2021; Searby et al., 2023). Mentoring has been shown to influence job satisfaction and retention of nurse practitioners, particularly when transitioning to the role (Horner, 2020). Despite this finding, to date there have been few formal mentoring programs for nurse practitioners in Australia, and none for AOD nurse practitioners.

## 2 BACKGROUND

Nurse practitioners have the capability to order advanced diagnostic tests, utilise clinical assessment and judgement to make working diagnoses, and prescribe medications (Nursing and Midwifery Board of Australia, 2021). In the AOD setting, nurse practitioners provide an ability to address prescriber shortages, particularly of opioid substitution medications such as methadone and buprenorphine, and provide holistic care for mental and physical ill health (Searby et al., 2023). Globally, shortages of skilled clinicians in AOD treatment has meant that individuals may encounter difficulty finding a prescriber of pharmacotherapy and skilled care for comorbidities occurring in the context of AOD use; arguments have been made that nurse practitioners provide a potential solution to healthcare resourcing to address these issues (Auty et al., 2020; Ellenbogen & Segal, 2020).

The global pool of nurse practitioners has grown immensely since the introduction of the role. At the last data count in Australia (2019), there were 1876 nurse practitioners, with 41 working in AOD roles (Australian Government Department of Health, 2020). Many of these nurse practitioners currently work in roles that provide primary care, harm reduction and consultation‐liaison services to people who use AODs, providing resources and care to a previously marginalised and underserved population. Although the services nurse practitioners provide are ideal to address the gap in healthcare provision for people who use AODs, several barriers to entry, uptake and full utilisation of nurse practitioners has been found in Australia (Searby et al., 2023).

Overall, the effectiveness of nurse practitioners has been explored to determine whether the role is suitable to provide autonomous healthcare services at a comparable or reduced cost to current modalities of care. For instance, Htay and Whitehead's (2021) systematic review of the comparisons between nurse practitioners and physician‐led care found that positive outcomes included greater patient satisfaction, better self‐management of chronic disease and better access to health advice. These findings are supported by a Cochrane review of nurse practitioner‐led care versus usual care (Laurant et al., 2018), which found similar outcomes in respect of patient satisfaction and health outcomes, however both of these reviews note the limitations of low quality evidence and call for a greater number of randomised control trials to improve the quality of evidence for nurse practitioner led care.

Comparisons between care as usual and nurse practitioner led care in AOD treatment are rare. However, studies that explore the effectiveness of these models are emerging in Australia. For example, Strike et al. (2023) explored a nurse‐practitioner led long acting injectable buprenorphine clinic in rural Melbourne, finding that the ‘wrap around’ model of care provided by the nurse practitioner coincided with a reduction in the number of syringes dispensed, potentially indicating reduced injecting drug use. Patient satisfaction with nurse‐practitioner led services has also been explored, with qualitative work exploring a nurse‐practitioner led ambulatory detox service in Queensland, Australia noting that consumers of the service valued the time the nurse practitioner could spend with them, the improved access to the service and the flexibility of the office‐based approach (Searby et al., 2024). Exploratory research on mental health services led by nurse practitioners have also shown that these models integrate successfully among existing services and are viewed favourably by clinicians (Barraclough et al., 2016).

The cost benefit argument of nurse practitioner led services has been examined in a report commissioned by the Australian Government (KPMG, 2018). This report examined eight case studies of nurse practitioner‐led models of care, and although none of these models were AOD specific, the report did find that roles in aged care would save up to 5000 emergency department visits each year, financially saving $5.7 million dollars in treatment costs. The roles in primary care were also attributed to healthcare access improvements for 10,000 Australians. These findings indicate the financial savings from greater implementation of nurse practitioner roles could be significant.

Despite the emerging evidence for nurse practitioner‐led models of care, the transition from experienced registered nurse to nurse practitioner can be problematic. A concept analysis of literature related to nurse practitioner role transition conducted by Barnes (2015) found that stress and anxiety often accompanied a shift from ‘care provider to care prescriber,’ and the autonomy that came with this change led many to feel overwhelmed and inadequate. Initial feelings of fear, stress and anxiety have been reported during role transition in a variety of settings, including rural primary care (Owens, 2018) and acute care (Dillon et al., 2016). Furthermore, poor role transition has been linked to burnout (Chen & Lin, 2022).

However, mentoring programs have emerged to address the issues associated with transition from experienced registered nurse to nurse practitioner, both to create sustainability in the nurse practitioner workforce, and to provide a pathway to autonomous practitioner. Internationally, several nurse practitioner fellowship or residency programs exist, designed to provide a supported transition to autonomous practice (Mounayar & Cox, 2021). For example, evaluation of a residency program at the Veteran's Affairs program in the United States, conducted over 1 year, showed statistically significant improvements in mentor ratings for inter‐professional collaboration and shared decision making were noted (Rugen et al., 2018). However, despite calls for these programs to be developed in Australia for a long period of time, no formal programs currently exist; rather, a mentoring arrangement for a set number of hours in the chosen specialty is required (Lee & Fitzgerald, 2008; MacLellan et al., 2015).

Existing mentoring programs for advanced practice nurses have shown promise in improving transition to practice, clinical leadership and interdisciplinary collaboration. For instance, Leggat et al., 2015 conducted a mentoring program with 18 Australian nurse practitioner candidates, with post‐testing showing a perceived transformational leadership, including in clinical collaboration. Furthermore, a mentoring program conducted with 40 neonatal nurse practitioners showed completing the program resulted in improved job satisfaction scores and intention to stay in the nurse practitioner role (Moss, 2022).

### 2.1 The program

The Connecting, Coaching and Changing Lives (CCC) program was conceived by the Drug and Alcohol Nurses of Australasia (DANA) Nurse Practitioner Sub‐Committee as a mentoring and education program for both nurse practitioners new to AOD treatment, and those encountering high numbers of individuals who use AODs in their daily practice. The program used peer support and mentoring to assist individuals transition from experienced registered nurse to nurse practitioner, and was inspired by a need to address the confusion, doubt, loss and disorientation of role, responsibilities, knowledge and relationship change recognised in Duchscher (2009).

The aims of the program were threefold: (1) to provide participants with the opportunity to gain confidence and competency in AOD treatment modalities and to connect with expert AOD nurse practitioners, (2) to address the needs of participants in the provision of evidence‐based services to individuals who use AODs and (3) to successfully transition and develop participants through mentoring, peer collaboration, support and professional development opportunities within a formal mentoring and peer‐collaboration environment.

The program comprised six interactive educational webinars (on alcohol, opioids, cannabis, benzodiazepines, amphetamine‐type substances and treating hepatitis C in people who use drugs) facilitated by an experienced nurse practitioner, six peer support webinars conducted immediately after the educational webinars which gave participants the opportunity to discuss clinical scenarios and their own practice with the experienced nurse practitioner, and monthly small‐group mentoring, where participants met with and experienced nurse practitioner mentor with content determined by participants. The program commenced in February 2022, with the final education session held ‘face‐to‐face’ at the DANA Annual Conference in Adelaide in August. A final ‘wrap up’ session was held with participants in October 2022. After each education session, participants were required to complete online quizzes, based on clinical scenarios discussed in the webinars, to provide immediate formative feedback on learning.

### 2.2 Study aim

The aim of this study was to explore the effectiveness and acceptability a pilot AOD nurse practitioner mentoring program; in particular, whether the program influenced attitudes towards people who use AODs, the clinical competence of participants and to seek qualitative evaluation on perceptions and potential improvements to the program for future iterations. In this context, clinical competence is defined by the C‐SCOPE instrument administered to participants: a self‐reported competency rating of the ability to test, manage and treat hepatitis C among people who use drugs (Grebely et al., 2019).

## 3 DESIGN

This project used a mixed methods design. A pre‐survey was administered to obtain demographic information and to determine perceived confidence, broad clinical skill and attitude towards working with people who use AODs, with a post‐survey used to measure changes in perceived confidence and clinical skill. Qualitative interviews were conducted concurrently to seek in‐depth perspectives on the program, and to seek suggestions for improvement. The qualitative phase of the project was conducted using Qualitative Description, a naturalistic framework that presents qualitative findings in the language used by participants and employs a ‘low‐inference’ approach (Neergaard et al., 2009).

The decision to use a mixed‐methods approach was driven both by the small sample size of the project, and the innovative nature of the program under investigation. Given there were only 15 participants in the CCC program, we felt that a triangulation approach was needed to determine whether the program had an impact on individual practice. This approach allowed the investigation of subjective opinions on elements of the program beyond survey measurements, which would be difficult to generalise to the wider population given the small sample size (Tashakkori & Teddlie, 1998). Although quantitative results can be used to measure change among participants due to the program, the incorporation of a qualitative component allowed these results to be further explored. In addition, qualitative interviews allowed the investigation of concepts difficult to measure using surveys, such as relationships between participants and their impact on role, and the ‘community of practice’ nature of the program (Kettles et al., 2011).

The integration approach taken during this study was that of ‘weaving;’ where both the quantitative and qualitative data sources of the project were presented contiguously to provide a comprehensive overview of the CCC program, and to ascertain improvements to future iterations of the program (Skamagki et al., 2022). The semi‐structured interview guide was created using the concept of building, where the initial pre‐survey results were used to guide the development of qualitative questions (Fetters et al., 2013).

### 3.1 Participants

A purposive sampling approach was used to identify participants, who had all taken part in the initial CCC mentoring program. Inclusion criteria for this study were nurse practitioners who had completed the mentoring program, with those who had not taken part or ceased their involvement excluded from this evaluation project. Research contact was made by the first two authors (A.S. & D.B.), who had no prior relationship with potential participants, with the inclusion criteria being confirmed in the opening screen of the online surveys and verbally prior to participants commencing a qualitative interview.

The project was advertised to CCC participants through the DANA, the national professional association for AOD nurse practitioners. Invites were sent via the DANA Nurse Practitioner Sub‐Committee, the group responsible for the creation and operation of the pilot CCC program. Participants who chose to take part in this project were asked to consent via a Qualtrics survey page, which administered the pre‐ and post‐program survey and to provide their email address for research assistant follow‐up if they agreed to participate in a qualitative interview. A Participant Information Form was available to participants on the first page of the Qualtrics site. The second author (D.B.) contacted participants by return email to arrange a suitable time to conduct the interview.

### 3.2 Data collection

The first phase of the study used a survey methodology, delivered online using the Qualtrics survey platform (Qualtrics, Provo, UT). In this survey, demographic data was collected including gender, year of endorsement and work setting. We used questions from the Brief Substance Abuse Attitude Survey (Chappel et al., 1985; Russolillo et al., 2023), a seven‐point Likert scale to ascertain participant attitudes to working with people who use AODs, and common clinical tasks associated with the role of AOD nurse practitioner. The wording of the scale was changed to reflect contemporary terminology (i.e. ‘alcoholism’ to alcohol use disorder), with face validity checked with members of the DANA nurse practitioner sub‐committee as described in the Reliability and validity section of this paper. In addition, questions from the Drug Problems Perceptions Questionnaire were used, a five‐point Likert‐type scale that asks participants to rate their competence, knowledge and awareness of working with individuals who use illicit substances (Watson et al., 2007). As new nurse initiated treatments for hepatitis C have emerged, we used questions drawn from the large scale C‐SCOPE study, a multinational study exploring awareness and treatment of hepatitis C among prescribers of opioid agonist treatment in Australia, Europe, the United States and Canada to determine perceived competence in working with individuals with hepatitis C (Grebely et al., 2019; Lanini et al., 2016; Nelson et al., 2011). All three instruments have undergone testing for reliability and validity elsewhere. The survey questions used in this study are shown in Table 1.

**TABLE 1:** Survey questions.

Drug problems perceptions questionnaire (seven‐point Likert‐type scale)
1 = strongly disagree, 2 = disagree, 3 = somewhat disagree, 4 = neutral, 5 = somewhat agree, 6 = agree, 7 = strongly agree
I feel I have a working knowledge of alcohol and alcohol related problems.
2 I feel I have a working knowledge of drugs and drug related problems.
3 I feel I know enough about the causes of problematic alcohol use to carry out my role when working with people who drink alcohol.
4 I feel I know enough about the causes of substance use disorder to carry out my role when working with people who use drugs.
5 I feel I know enough about alcohol use disorder to carry out my role when working with people who drink alcohol.
6 I feel I know enough about substance use disorder to carry out my role when working with people who use drugs.
7 I feel I know enough about the psychological effects of alcohol to carry out my role when working with people who drink alcohol.
8 I feel I know enough about the psychological effects of drugs to carry out my role when working with people who use drugs.
9 I feel I know enough about the factors which put people at risk of developing alcohol use disorder to carry out my role when working with people who drink alcohol.
10 I feel I know enough about the factors which put people at risk of developing substance use disorder to carry out my role when working with people who use drugs.
11 I feel I can appropriately advise my patients about drinking alcohol and its effects.
12 I feel I can appropriately advise my patients about drugs and their effects.
13 I feel I have the right to ask patients about their alcohol consumption where necessary.
14 I feel I have the right to ask patients about their drug use where necessary.
15 I feel I have the right to ask a patient for any information that is relevant to their alcohol consumption.
16 I feel I have the right to ask a patient for any information that is relevant to their drug use.

Brief substance abuse attitude survey (five‐point Likert‐type scale)
1 = strongly disagree, 2 = disagree, 3 = undecided, 4 = agree, 5 = strongly agree
17 Alcohol or substance use disorder is associated with a weak will.
18 Nurses who detect problematic alcohol and/or substance use disorder early improve the chance of treatment success.
19 A person with an alcohol or substance use disorder who has relapsed several times probably cannot be treated.
20 Long‐term outpatient treatment is necessary for the treatment of alcohol or substance use disorder.
21 Abstinence is a necessary goal in the treatment of alcohol or substance use disorder.
22 Alcohol use disorder is a treatable illness.
23 Substance use disorder is a treatable illness.
24 A hospital is the best place to treat a person with an alcohol or substance use disorder.
25 Most alcohol and drug dependent persons are unpleasant to work with as patients.
26 Coercive pressure, such as threat or punishment, is useful in getting resistant people to accept treatment.

C‐SCOPE measures of competence in managing hepatitis C (seven‐point scale)
1 = none or no skill at all, 2 = vague knowledge, skills or competence, 3 = slight knowledge, skills or competence, 4 = average among my peers, 5 = competent, 6 = very competent, 7 = expert, teach others
27 Ability to ensure people at risk of hepatitis C infection are regularly screened.
28 Ability to interpret hepatitis C test results and diagnose hepatitis C.
29 Ability to assess severity of liver disease in patients with hepatitis C.
30 Ability to advise patients about new therapies for hepatitis C.
31 Ability to educate clinic staff about hepatitis C and to serve as a contact point for questions or issues.
32 Knowledge of new treatments/regimens for hepatitis C.
33 Ability to treat hepatitis C patients and manage side effects

The second phase of the study used a semi‐structured interview process to gain in‐depth qualitative data from participants, particularly their perceptions of the CCC program and to explore any changes in perceived confidence and skill noted in the first phase of the study. Interviews were conducted by a research assistant with experience in conducting qualitative studies (D.B.). All interviews were conducted by telephone, and participants did not receive compensation, reward, or reimbursement for participating. Creation of the semi‐structured interview tool as guided by the initial survey results and formulated to further explore perceived confidence and competence in the core roles as an autonomous nurse practitioner. The questions further probed the impact of the CCC program on these capabilities. Finally, suggestions for improvement of the program were sought from participants. All interviews were audio recorded and transcribed verbatim by a professional transcription agency for analysis, with the mean duration of interviews 32:36 (SD 7:42).

### 3.3 Data analysis

Survey data were analysed using SPSS version 29 (IBM Corporation, Armonk, NY). We used inferential statistics to compare pre‐ and post‐survey responses, using a paired samples *t* test after removing incomplete responses (Xi et al., 2018). Given the small sample size and missed responses in the post‐test, we treated these results as exploratory and indicative rather than having the statistical power to draw conclusions. Qualitative data was analysed using structural coding, a method of data analysis used to collate, index and label data relevant to a specific analysis (Saldaña, 2013). In this case, we used structural coding to analyse the data in accordance with the aims of the study (an exploration of the pilot nurse practitioner mentoring program).

### 3.4 Ethical considerations

This study as reviewed and approved by the relevant university ethics committee as a low‐risk project (reference number HEAG 17_2022) prior to data collection commencing. All participants provided consent on the online survey, and further verbal consent prior to the interviews commencing.

### 3.5 Validity and reliability

Validity and reliability for the three quantitative instruments has been tested extensively. The Substance Abuse Attitude Survey was tested for reliability and validity by being scored by clinicians experienced in AOD treatment (*n* = 116), finding internal consistency over repeated administrations (Chappel et al., 1985). Similar results were found when validating the instrument with 598 college students (Jenkins et al., 1990). The Drug Problems Perceptions Questionnaire was tested for validity and reliability with a stratified random sample of clinicians (*n* = 672) in mental health and AOD services in the United Kingdom, finding reliability, internal consistency and relevance in terms of conduct validity in testing (Watson et al., 2007). The C‐SCOPE instrument measuring clinical competence in Hepatitis C treatment was based on questions used in previously validated studies, with a regression model used to evaluate competency endpoints (Grebely et al., 2019).

Face validity of the survey was assessed by members of the DANA nurse practitioner sub‐committee, which comprises expert nurse practitioners. Given the instruments used in the survey have undergone testing for reliability and validity elsewhere, we asked the committee to focus on question meaning, especially where terms had been changed to reflect contemporary terminology (i.e. ‘alcoholism’ to alcohol use disorder). To further assess survey validity, we conducted a pilot with members of the DANA management committee, seeking verbal feedback on the structure of the survey and flow of the questions asked.

This study was reported in accordance with the Good Reporting of a Mixed Methods Study (GRAMMS) checklist (O'Cathain et al., 2008).

## 4 FINDINGS

### 4.1 Survey findings

Fifteen participants took part in the pre‐program survey. Participants were predominantly female (*n* = 12, 80%) and working as a nurse practitioner (60%). Participants had a high number of years of overall nursing experience (M = 22, SD = 8.3), but a low number of years as nurse practitioners (M = 1.5, SD = 3, range 0–10 years) eight participants reported having no experience practicing as endorsed nurse practitioners. Most participants (*n* = 6, 40%) reported AOD (AOD) treatment services as their primary place of employment, followed by community health services (*n* = 3, 20%), and most resided in the Australian state of Victoria (*n* = 7, 46.7%), followed by Queensland (*n* = 6, 40%). Participants had a mean age of 46.3 years (SD = 8.4). The full demographic breakdown of participants is shown in Table 2.

**TABLE 2:** Participant demographic characteristics.

Characteristics	Sample	*M*	*SD*
Gender			
Male	3 (20%)		
Female	12 (80%)		
Age		46.3	8.4
Total nursing experience (years)		22	8.3
Experience as a nurse practitioner (years)		1.5	3
Registration status			
Nurse practitioner	9 (60%)		
Nurse practitioner candidate	6 (40%)		
Highest level of qualification			
Graduate certificate	1 (6.7%)		
Graduate diploma	1 (6.7%)		
Masters degree	12 (80%)		
Doctor of Philosophy (PhD)	1 (6.7%)		
Main work setting			
Alcohol and other drug (AOD) treatment service	6 (40%)		
Community health service	3 (20%)		
Consultation‐liaison	1 (6.7%)		
Emergency department	1 (6.7%)		
Mental health (community)	1 (6.7%)		
Prison/correction or detention centre	1 (6.7%)		
Other^a^	2 (13.3%)		
Employment status			
Full time	7 (46.7%)		
Part time	8 (53.3%)		
State of employment			
New South Wales	1 (6.7%)		
Queensland	6 (40%)		
Victoria	7 (46.7%)		
Western Australia	1 (6.7%)		

^a^ Other comprised primary care mental health and addiction, and private practice telehealth.

Participants completed three measures on the survey, as outlined in the methods section of this paper, at two time points: once before the commencement of the CCC program, and once immediately after the final session. As shown in Table 3, participants rated themselves as being highly skilled in many facets of care for people who use AODs, and average Likert‐type scores indicate that participants perceived themselves as being non‐judgemental. Furthermore, the scores indicate that participants were likely to feel that enquiring about AOD use was part of their role and were willing to have these clinical discussions.

**TABLE 3:** Pre‐ and post‐survey scoring.

Question item	*n* (pre‐test)	Pre‐test mean (SD)	*n* (post‐test)	Post‐test mean (SD)	*p*‐value	*t*‐statistic
Drug problems perceptions questionnaire (seven‐point Likert‐type scale) 1 = strongly disagree, 2 = disagree, 3 = somewhat disagree, 4 = neutral, 5 = somewhat agree, 6 = agree, 7 = strongly agree						
I feel I have a working knowledge of alcohol and alcohol related problems.	15	6.00 (1.21)	10	6.40 (0.70)	0.172	1.000
2 I feel I have a working knowledge of drugs and drug related problems.	15	6.00 (1.21)	10	6.40 (0.84)	0.296	0.557
3 I feel I know enough about the causes of problematic alcohol use to carry out my role when working with people who drink alcohol.	15	6.00 (1.26)	10	6.30 (0.82)	0.041	1.964
4 I feel I know enough about the causes of substance use disorder to carry out my role when working with people who use drugs.	15	5.87 (1.20)	10	6.40 (0.84)	0.500	0.000
5 I feel I know enough about alcohol use disorder to carry out my role when working with people who drink alcohol.	15	5.93 (1.24)	10	6.40 (0.84)	0.296	0.557
6 I feel I know enough about substance use disorder to carry out my role when working with people who use drugs.	15	5.87 (1.20)	10	6.40 (0.70)	0.500	0.000
7 I feel I know enough about the psychological effects of alcohol to carry out my role when working with people who drink alcohol.	15	5.73 (1.24)	10	6.40 (0.70)	0.500	0.000
8 I feel I know enough about the psychological effects of drugs to carry out my role when working with people who use drugs.	15	5.80 (1.22)	10	6.40 (0.70)	0.500	0.000
9 I feel I know enough about the factors which put people at risk of developing alcohol use disorder to carry out my role when working with people who drink alcohol.	15	5.80 (1.17)	10	6.50 (0.71)	0.172	−1.000
10 I feel I know enough about the factors which put people at risk of developing substance use disorder to carry out my role when working with people who use drugs.	15	5.80 (1.17)	10	6.50 (0.71)	0.172	−1.000
11 I feel I can appropriately advise my patients about drinking alcohol and its effects.	15	5.87 (1.20)	10	6.50 (0.71)	0.296	−0.557
12 I feel I can appropriately advise my patients about drugs and their effects.	15	5.80 (1.17)	10	6.50 (0.71)	0.172	−1.000
13 I feel I have the right to ask patients about their alcohol consumption where necessary.	15	6.27 (1.00)	10	6.60 (0.97)	0.339	0.429
14 I feel I have the right to ask patients about their drug use where necessary.	15	6.27 (1.00)	10	6.50 (0.97)	0.172	1.000
15 I feel I have the right to ask a patient for any information that is relevant to their alcohol consumption.	15	6.07 (1.00)	10	6.30 (1.06)	0.222	0.802
16 I feel I have the right to ask a patient for any information that is relevant to their drug use.	15	6.00 (0.97)	10	6.20 (1.14)	0.254	0.688
Brief substance abuse attitude survey (five‐point Likert‐type scale) 1 = strongly disagree, 2 = disagree, 3 = undecided, 4 = agree, 5 = strongly agree						
17 Alcohol or substance use disorder is associated with a weak will.	15	1.20 (0.40)	10	1.20 (0.40)	0.084	−1.500
18 Nurses who detect problematic alcohol and/or substance use disorder early improve the chance of treatment success.	15	4.20 (1.05)	10	4.30 (1.19)	0.007	−3.000
19 A person with an alcohol or substance use disorder who has relapsed several times probably cannot be treated.	15	1.13 (0.34)	10	1.30 (0.46)	0.041	−1.964
20 Long‐term outpatient treatment is necessary for the treatment of alcohol or substance use disorder.	15	2.73 (1.00)	10	3.00 (1.55)	<0.001	9.000
21 Abstinence is a necessary goal in the treatment of alcohol or substance use disorder.	15	2.13 (1.02)	10	1.30 (0.46)	0.18	2.449
22 Alcohol use disorder is a treatable illness.	15	4.67 (0.47)	10	4.30 (1.19)	0.278	0.612
23 Substance use disorder is a treatable illness.	15	4.67 (0.47)	10	4.70 (0.46)	0.084	−1.500
24 A hospital is the best place to treat a person with an alcohol or substance use disorder.	15	1.73 (0.44)	10	1.40 (0.49)	0.084	1.500
25 Most alcohol and drug dependent persons are unpleasant to work with as patients.	15	1.47 (0.81)	10	1.20 (0.40)	0.084	−1.500
26 Coercive pressure, such as threat or punishment, is useful in getting resistant people to accept treatment.	15	1.07 (0.25)	10	1.10 (0.30)	0.172	−1.000
C‐SCOPE measures of competence in managing hepatitis C (seven‐point scale) 1 = none or no skill at all, 2 = vague knowledge, skills or competence, 3 = slight knowledge, skills or competence, 4 = average among my peers, 5 = competent, 6 = very competent, 7 = expert, teach others						
27 Ability to ensure people at risk of hepatitis C infection are regularly screened.	15	5.07 (1.06)	10	5.60 (0.70)	0.500	0.000
28 Ability to interpret hepatitis C test results and diagnose hepatitis C.	15	4.13 (1.26)	10	5.40 (0.70)	0.007	−3.000
29 Ability to assess severity of liver disease in patients with hepatitis C.	15	4.13 (1.02)	10	5.00 (0.94)	0.097	−1.406
30 Ability to advise patients about new therapies for hepatitis C.	15	4.40 (1.08)	10	5.80 (0.63)	<0.001	−5.014
31 Ability to educate clinic staff about hepatitis C and to serve as a contact point for questions or issues.	14	4.21 (1.06)	10	5.50 (0.85)	<0.001	−6.708
32 Knowledge of new treatments/regimens for hepatitis C.	15	4.07 (0.91)	10	5.50 (0.85)	<0.001	−6.708
33 Ability to treat hepatitis C patients and manage side effects	15	3.87 (1.18)	10	5.30 (0.95)	<0.001	−9.000

Eleven participants completed the post survey, and scores remained relatively stable in most domains, with statistically significant changes noted in three of the first 16 survey items. These items were the questions ‘I feel I know enough about the causes of problematic alcohol use to carry out my role when working with people who drink alcohol,’ (*t*(9) = 1.964, *p* = 0.041), ‘Nurses who detect problematic alcohol and/or substance use disorder early improve the chance of treatment success,’ (*t*(9) = −3.000, *p* = 0.041), and ‘Long‐term outpatient treatment is necessary for the treatment of alcohol or substance use disorder,’ (*t*[9] = 9.000, *p* < 0.001). Conversely, the final seven question items related to the treatment and knowledge of hepatitis C showed statistically significant changes in five question items, indicating that the CCC program increased knowledge and skill in the management of hepatitis C. All five survey questions that recorded a statistically significant change between pre‐ and post‐program testing had a mean scale rating of ‘competent.’

### 4.2 Qualitative findings

Ten participants of the program took part in a qualitative interview. Most (*n* = 9) were female and working as endorsed nurse practitioners (*n* = 8), with the remaining two participants being nurse practitioner candidates. Five were from the Australian state of Victoria, three from New South Wales and two from Queensland. As described in the Methods section of this paper, the semi‐structured interview guide was developed to explore quantitative results obtained from the pre‐ and post‐test survey to explore responses, an important step given the small sample size and inability to extrapolate the statistical results alone. Using the structural coding approach outlined in the design section, we analysed data using four broad themes: clinical learnings from the program, the influence of the program on work practices, support gained from the program and suggested improvements to the program.

### 4.3 Clinical learnings from the program

The first theme explored during the interview process was to determine whether participants took any clinical learnings from the program. As described earlier, the format of the program involved an online educational session with the presentation of case studies to apply learnings. Overall, responses to the value of this content ranged from consolidation of existing knowledge, to improving the knowledge base of participants, particularly where their work with individuals who use AODs was limited:

Overall, it improved my knowledge base and provided me with more confidence to be able to treat clients. (Participant 5. NP)

A focus on hepatitis C was included in the program due to the prevalence of this disease, particularly among people who use illicit and injected drugs. Participants described the focus on hepatitis C as being valuable:

… I feel much more confident being able to initiate treatment. Because I wasn't doing Hep C treatment previously and so, since I've attended this, and obviously alongside of it I've also done additional education as well, but certainly this has got me on the path of feeling comfortable and confident to be able to prescribe and initiate Hep C treatment. (Participant 10, NP)

Participants who recognised that the hepatitis C management and treatment focus also identified that these learnings were applicable to their own practice:

I think perhaps the one thing that I've most been able to improve within my own workplace and my own practice is starting that treatment journey for someone who's facing a new diagnosis of Hepatitis C or actually having a reoccurring infection of Hepatitis C. (Participant 6, candidate)

Several participants also spoke of the program as not only providing education in a diverse range of topics related to AODs, but also of the ability to learn from several experienced nurse practitioners. As the following participant mentions, these skills were often considered ‘core,’ but the value of the program lay in the diverse perspectives that were available:

I found it is a refresher in some aspects of the pilot that I was participating in because they're our core skills that I was employed to do anyway, but it definitely helped in relation to having different perspectives from the experience of practitioners. (Participant 4, NP)

Although participants described the theoretical component of the course as valuable, some did find a disconnect between theory and application to clinical practice. The concept of being a ‘novice’ nurse practitioner was expressed by several participants, often leading to comparisons with more experienced peers:

The resources within the triple C program are very comprehensive. I have an excellent resource to refer to. So in that theoretical space I feel more confident. In the practical clinical space I still sit sort of around the novice space. Sort of more than a novice but certainly not anywhere near my peers who are working in this space. (Participant 2, NP)

However, some participants described a ‘consolidation’ of their existing knowledge, confirming that their practice was evidence‐based and correct. As the following example indicates, the linkage between educational content and mentor allowed them to apply the learnings to practice:

I think it just consolidated what I was doing. I've also since gained a second part‐time position working for an Aboriginal and Torres Strait Islander organisation and certainly some of the information I gained from my, another mentee in the group and also from the education sessions I've been able to put into practice in that role. (Participant 6, candidate)

Furthermore, consolidation of the clinical content with participant's current practice reinforced that their practice was contemporary, as illustrated by the following quote:

I think because of feeling more confident again, yes, feeling that ‘Oh yep, I'm current,’ and even if there's other people around that I can ask. I can feel confident that I can actually do that, if that make sense. (Participant 8, NP)

As identified by the following participant, the educational sessions and case studies provided alternative perspective and ‘options’ for different treatment and care decisions:

… it gave me different options that I didn't know were around out there, and hearing how like in the case studies, how people managed particular clients, was very helpful I found. It gave you a rounder idea of how you would support somebody in that situation and some of the cases were quite complicated, so to see how they managed that was good. It gave you an idea of what treatment looked like I suppose. (Participant 9, NP)

In summary, participants described the clinical education component of the CCC program as valuable, particularly if they had limited exposure in providing diagnosis, treatment and management of individuals who used AODs. Further, participants described this learning as a form of consolidation of their existing knowledge base, and a confirmation that their practice was contemporary and evidence based.

### 4.4 The influence of the program on work practices

The second theme explored during the interview process was the influence of the program on participant work practices. As noted in the previous theme, participants recognised that the learnings were valuable to expand and consolidate existing knowledge, however in this theme we sought to determine whether the CCC program was transferable to clinical workplaces. Several participants noted that the program provided them with knowledge specific to AODs, often an area where participants described their clinical knowledge as wanting. As noted by the following participant, the learnings provided during the program provided a ‘refresher’ for those who had not worked in the area for some time:

From my perspective, I had been out of drug and alcohol for about 5 years, working in mental health. So, coming back, it was almost timely that the program started … this was a good refresher for me to attend. (Participant 1, NP)

The program also allowed participants to compare their practice with other nurse practitioners who were experienced and established in the AOD treatment field. It also allowed exposure to different models of care, including where experienced nurse practitioners had developed their own models of care:

I think it was a great experience to see where other nurse practitioners are working and what their model of care looks like. One of the other mentors has a role in a telehealth withdrawal service for rural and remote community and … he works for a [non‐government organisation], so looking at how he has developed model of care and business model that works for him. (Participant 6, candidate)

Following on from the previous theme, participants also described the program as having an influence on their ‘clinical currency,’ particularly when discussing models of care among the group. As the following narrative indicates, the program was useful to remain current in contemporary, evidence‐based practice that participants may not otherwise have the opportunity to research:

… to know that I am current, and I am up‐to‐date … because that can sort of sit by the wayside of your goals, and you might be using things that, ‘Oh, actually that isn't current,’ and things change all the time. For example, there's different Morphine injectables that's been out in the last 12 or 18 months. That's changed what I was able to prescribe a few years ago. So, ensuring that you've got updated knowledge, you know, the Hep C treatment has changed all the time in terms of how we screen, what we need, and then how we prescribe, and so that's nice to know … it makes sure that you are up‐to‐date and current in your practice which is really important. (Participant 8, NP)

Participants also noted that the program reinforced the concept of holistic care, rather than providing a course that focused purely on diagnostics and prescribing. Participants noted that the concept of providing care beyond alcohol or other drug use, particularly psychosocial care, was a topic embedded in the CCC program:

I often give the example, you know, people will compare a nurse practitioner to a doctor … I say, ‘Well, you know, you can't judge all doctors, but they are trained in the medical model of care,’ which it predominantly is. ‘What's brought you into the room today? Ah, you've got a broken foot. Let's fix the broken foot, and then off you go.’ However, as a nurse, somebody would come into the room and they've got a broken foot, ‘Ah, okay, so you have anybody at home that can help you get around? Like, how did you get here today? Do you have a children that need to go to school? How are you going to get food in the house if you live on your own?’ I think we look at the much broader area of somebody's life. Like, the illness or disease is part of their life, it's not just there sitting on the side on its own. (Participant 8, NP)

Finally, the CCC program was described as clinically relevant by participants as it allowed them to expand on areas in their clinical practice they felt were missing. For instance, the following account indicates that a perceived area of opportunity to provide more comprehensive care was a key driver for participation in the course:

I'd really like to do more dual diagnosis because I feel that's a really big area that's missing. We sort of work in a side of health where it's either mental health or drug and alcohol, but I'd really like to be able to combine those positions because a lot of the clients that I see have both mental health and drug and alcohol and we're trying to meet with drug and alcohol services to make a bit of a better relationship. But that would be my dream, to be able to be a dual diagnoses nurse practitioner and part of the reason why I did the course. (Participant 9, NP)

### 4.5 Support gained from the program

The support provided by the program was the most frequent benefit mentioned by interview participants. Support took the form of mentoring groups, and to a lesser extent, discussion when case studies were presented to the group. As outlined in the Background section of this paper, mentoring sessions were run in a small group format, with an experienced nurse practitioner guiding the discussion. Participants considered this format a substantial advantage of the program:

It was good being with people who were very experienced as well … I've sort of been aware of drug and alcohol within my training, but not to the extent of this program. I probably just found it really exciting, and it was so nice being with other nurse practitioners. (Participant 9, NP)

Participants spoke of the ongoing relationships formed during the program, including connections made and the intention to keep these relationships going into the future. Some participants also noted that the CCC program gave them the opportunity to have collegial relationships with other nurse practitioners, which was not always possible in their work setting:

I now have a network of other nurse practitioners that we all have our contact details, and they've said, ‘You can call on us if you want any advice’. I'm more aware of where to look for policies, procedures. Being in a rural area, you are a bit isolated, and talking with people in different states, cities, towns and hearing about what they're doing, I guess, it improves my knowledge of what best practice is and what new practices are out there, because, I don't know, I think, being a small country hospital, we don't have research projects going. (Participant 3, candidate)

Participants noted that the support and mentoring component of the program allowed closer discussion of the case studies, which the following participant notes gave a different perspective on clinical decision making:

There was a different topic each month and then it was back put through the mentoring support as well, which gives the opportunity to talk about deidentified information around potential practice issues or scenarios, which I found really helpful to get a different set of eyes or ears on something. So, it helped me look at something in a different way. (Participant 4, NP)

As noted in previous research (Searby et al., 2023), a number of participants commented on the delay between finishing their educational program to become a nurse practitioner and either becoming endorsed or finding a position. As the following participant indicates, the program provided confidence in transitioning to the role of nurse practitioner:

I do feel, I still, and this is I think just in the delay in my finishing my Masters and starting the nurse practitioner role. I finished my Masters, ready to go August last year, endorsed, ready to go and then I've had to wait right up until three weeks ago for my position … I lost a bit of confidence which is where the Triple C program was perfect for me because it kept that learning happening and that network of other people in. (Participant 7)

During all interviews, participants spoke highly of the support gained from the program. Several spoke to this support as being ongoing and necessary to function as an autonomous practitioner, with the ability to find these supportive relationships difficult in some practice settings. To this end, the program was an effective ‘community’ for nurse practitioners:

I think it's really important, particularly as a nurse practitioner … we're expected to have clinical expertise at the highest level of nursing. But a lot of us … sadly, nurse practitioners are not often able to support each other in that private practice setting, I think making sure that you do have time to spend time with other colleagues is really important. (Participant 8, NP)

The mentoring component of the program was also described as an opportunity to discuss commencing as a nurse practitioner, particularly regarding logistical issues in the journey to autonomous practitioner. As the following account indicates, the mentoring sessions were an opportunity to discuss aspects such as technology access and applying for a provider number to commence billing as a nurse practitioner:

Look, if the biggest influence of the CCC program was the mentoring side of it, that there was people in similar situations to ourselves. There's lots of things that they don't teach you at university, like getting a provider number, the odds of getting into PRODA [a healthcare provider digital access platform] … How do you go about billing and NBS funding and all that sort of stuff. They don't teach that at all at university, so having people in the same place as yourself with that was really beneficial to me. (Participant 10, NP)

In summary, the mentoring component was described by participants as the most valuable part of the CCC program. The ability to network with colleagues, and to learn from experienced nurse practitioners who had ‘walked the path’ from registered nurse, to transitioning to autonomous, advanced practitioners was described as invaluable to participants.

### 4.6 Suggested improvements to the program

The final theme explored during qualitative interviews was suggested improvements to the program. Given the CCC program was a pilot, we sought to solicit comments on how the program could improve in future iterations. Although several participants had forged ongoing relationships, one participant recommended that a formalised system of ongoing communication and support was established:

The only thing I'd like to see is maybe you have an alumni thing for people that have completed it stay in touch, but our group's doing that anyway, but maybe even a formal thing. (Participant 3, candidate)

As evident in the last theme, participants who were new nurse practitioners requested support with the practical aspects of their transition to autonomous practitioner. While some received this, some participant feedback requested a greater focus on practicalities rather than the theory of treatment and assessment:

Some of the practical things that the practitioners need in order to practice when they first qualify, like Medicare prescriber number, provider numbers, those sorts of things, they're all foreign to us. At our end you don't see that really. So, all that initial Q&A type stuff of what you need to know as a new nurse practitioner. (Participant 4, NP)

A solution to address this was offered by one participant, who suggested that mentoring groups were allocated based on experience to allow time for those new to the role to discuss their transition with experienced nurse practitioners:

I notice, yeah, one of the suggestions also that come out of our follow‐up was around is people like me who are very, very new to NP roles but had experience and then there were others that have been NPs for a certain amount of time and so whether the mentoring groups needed to be, you know, sort of grouped into experience rather than, yeah, rather than sort of randomly… yeah. (Participant 7, NP)

Another participant noted that the case studies were sometimes quite complex, with clinical situations that were potentially beyond the realm of new nurse practitioners. These accounts add weight to the need to stratify mentoring and learning groups depending on the stage of the participant's career:

I think again even the case studies that they provided, I would be probably even too uncomfortable to treat those clients, so I think the big mistake they have in the CCC program is they made it too complex for the cohort they were teaching … a lot of them were people I would refer to a doctor or I would refer to an addiction specialist because I'd be too uncomfortable as a nurse practitioner to do their treatment. So, in general, normal clients, yeah, I'd be happy to commence treatment if I was allowed, but the clients represented in the CCC program are just a bit too much. (Participant 5)

Although several jurisdictional differences exist between states and territories in Australia, participants did not recommend that groups were based on regions. Participant comments recognised the value in learning from peers who may be in a different state or territory, however as the following participant notes, there would be value in a mechanism to ask questions from practitioners in the same state or territory:

Just one more thing while I think about it is, whether or not it would be helpful to have a State‐based group because our group was obviously, the peer support group, they were mixed up. So, you had obviously, Victoria, Queensland, New South Wales and I mean, I see value in that because I didn't know anyone else really in my group, but then I also see the flipside is sometimes if I had a question or a query and I asked about, which may have been say a legal query or for instance, can a nurse practitioner in New South Wales prescribe medicinal cannabis? But because everyone's from different States, everyone had a different answer. (Participant 1, NP)

The responses to a request for improvements were constructive and recognised the nature of the program as a first‐run pilot. Most of these comments were qualified with participant satisfaction with the program, and reiterated the value participants felt the program had on their careers.

## 5 DISCUSSION

In this paper, we aimed to explore a pilot mentoring program for nurse practitioners. To our knowledge, this program, specific to AOD nurse practitioners or those working with individuals who use AOD, is the first of its kind in Australia. As outlined earlier, the program was conceived to address issues inherent in nurse practitioner transition, including a perceived lack of support from experienced peers, that impact the successful movement from advanced registered nurse to autonomous nurse practitioner (Faraz, 2016).

Our survey results indicate that participants in the program held a high perceived level of confidence to work with individuals who use AODs. Furthermore, they held positive therapeutic attitudes towards individuals who were either seeking alcohol and/or other drug treatment, or who used alcohol and/or other drugs comorbidly with other health conditions. These results likely reflect the motivation of individuals who take up specific AOD nursing positions, or those where working with individuals who use alcohol and/or other drugs is a substantial portion of their workload. Searby et al. (2023) noted in a mixed‐methods study of Australian AOD nurse practitioners that many described their motivation to move to the nurse practitioner position as being a logical extension of their advanced practice role and skills, hence potentially explaining the high levels of self‐reported confidence and competence in working in this space.

Although based on a small sample size and therefore not conclusive, the survey results do indicate that the inclusion of hepatitis C content did indicate a self‐reported increase in the willingness and competence in diagnosing and managing hepatitis C in individuals who use AODs. Similar programs have shown good results in engaging nurses in providing care and treatment for hepatitis C, for example Lobo et al.'s (2015) program in Western Australia, where hepatitis nurses were key to providing treatment uptake and access. Given the demonstrated effectiveness of nurse led hepatitis models of care among marginalised populations such as prisoners (Lloyd et al., 2013; Papaluca et al., 2019), the increase in knowledge and confidence among our participants is promising and warrants further research to determine whether clinical practice and treatment offerings have been influenced by the CCC program.

In our qualitative interviews, participants described mentoring and peer connection as the greatest advantage of the program, particularly when they were starting out as neophyte nurse practitioners. The ability for participants to connect with experienced practitioners for support with the fundamental tasks of becoming an autonomous nurse practitioner was rated highly by participants, with several describing the mentoring relationship as ongoing. Although scant research exists on nurse practitioner mentoring, a qualitative study of 16 Australian nurses identified that strong support networks were key to improving workplace resilience (McDonald et al., 2016). Furthermore, a survey study of 3983 Australian nurses working with older adults with dementia found that mentoring was critical to retention, indicating that support is essential for nurses working with complexity, such as the nurse practitioners that took part in the CCC mentoring program.

Although this program does not replace the residency programs that involve an internship and formalised support structure, our results do indicate that nurse practitioners value peer support and ‘communities of practice’ facilitated by experienced nurse practitioners. In contrast, residency programs are often run by organisations with the intention to improve the transition experience and increase retention of advance practice nurses, although the exact composition and content of programs remains diverse (Kesten et al., 2021; Martsolf et al., 2017). Although formalised residency programs are not widespread in Australia, the exploratory results gained from participants in the CCC program does provide a basis for the investigation of formalised programs in future.

### 5.1 Limitations

To our knowledge, this is the first Australian study to explore a novel mentoring program for nurse practitioners working with people who use AODs, however there are limitations that need to be considered when interpreting our results. The study relied on a small sample size, and although we had a good response from most participants of the CCC program, the post‐test survey response rate was smaller than the pre‐test. Given the small sample size in the survey component of the study, the quantitative results should be treated as exploratory, with repeated measures taken over several iterations of the CCC program to reliably determine whether there is an effect. In addition, the survey results rely on self‐rated measures of competency rather than objective measures such as observation, and only measured specific clinical skills in hepatitis C management, diagnosis and treatment. This was a deliberate decision as treatments for hepatitis C are emerging as nurse initiated and driven, especially for nurse practitioners in the AOD treatment space (Harney et al., 2021; Nario et al., 2021). However, future research on programs such as this could measure perceived clinical competence in domains such as diagnosis and prescribing.

Furthermore, our methodology did not allow us to determine whether changes persisted beyond the completion of the program. Future research on programs of this nature should consider adding a time‐point beyond the conclusion of the program to determine whether changes in practice described in both phases of the research translate to clinical practice and are sustained over time. Finally, our qualitative results describe the subjective experiences of participants in the CCC program and should not be considered representative of all nurse practitioners completing mentoring programs.

## 6 CONCLUSION

Nurse practitioners face many challenges when transitioning to autonomous practice, impacting retention and sustainability of advanced practice nursing roles. Our study, exploring a pilot mentoring program for nurse practitioners working with individuals who use AODs, indicates that although clinical learnings geared towards complex presentations were welcome, the support provided by peers and mentors was highly valued by participants. Several of these relationships were reported to continue beyond the completion of the program, providing an informal ‘community of practice’ for participants to obtain clinical support and guidance. Although we noted some improvements in perceived competency and confidence in providing care and treatment to individuals who use AODs, the small sample size and decline in post‐test responses mean these findings are indicative rather than conclusive. Future evaluation of nurse practitioner mentoring programs, conducted outside formal residency or internship programs, is required to determine the effect on clinical confidence and competence.

## 7 RELEVANCE TO CLINICAL PRACTICE

Although some jurisdictions have moved towards residency and internship programs to support nurse practitioner transition to practice, there are many countries around the world where the specialisation and small number of nurse practitioners have not made these programs feasible. This study explored a pilot program designed to provide specialist education and support to nurse practitioners working in either AOD use treatment, or with individuals who use alcohol and/or other drugs. Our findings indicate that although there were some improvements in confidence and competence in domains of treatment, participants valued peer support and the ability to be mentored by experienced nurse practitioners. We recommend further investigation of nurse practitioner mentoring programs, conducted outside formal hospital settings, to address retention issues occurring during the transition from advanced practice nurse to autonomous nurse practitioner.

## AUTHOR CONTRIBUTIONS

All authors have agreed on the final version and meet at least one of the following criteria recommended by the CRediT contributor roles taxonomy. AS: conceptualization, methodology, investigation, formal analysis, project administration, supervision, writing (original draft). DB: conceptualization, investigation, data curation, formal analysis, project administration, writing (original draft). CB: conceptualization, validation, project administration, resources, writing (review and editing). JH: conceptualization, validation, resources, writing (review and editing). DS: conceptualization, validation, resources, writing (review and editing).

## FUNDING INFORMATION

No external funding was received for this study.

## CONFLICT OF INTEREST STATEMENT

The authors have no conflict of interest to declare.

## Data Availability

The data that support the findings of this study are available on request from the corresponding author. The data are not publicly available due to privacy or ethical restrictions.
